# Basic critical care echocardiography training of intensivists allows reproducible and reliable measurements of cardiac output

**DOI:** 10.1186/s13089-019-0120-0

**Published:** 2019-04-16

**Authors:** Christian Villavicencio, Julen Leache, Judith Marin, Iban Oliva, Alejandro Rodriguez, María Bodí, Nilam J. Soni

**Affiliations:** 1 Critical Care Department, Joan XXIII-University Hospital, Mallafre Guasch 4, 43007 Tarragona, Spain; 2 Critical Care Department, Hospital del Mar-Research Group in Critical Illness (GREPAC), Institut Hospital del Mar d’investigacions Mèdiques (IMIM), Barcelona, Spain; 3 Division of Pulmonary & Critical Care Medicine, University of Texas Health San Antonio, San Antonio, TX USA; 4 Division of General & Hospital Medicine, University of Texas Health San Antonio, San Antonio, TX USA; 5 Section of Hospital Medicine, South Texas Veterans Health Care System, San Antonio, TX USA

**Keywords:** Pulmonary artery catheter, Critical care echocardiography, Cardiac output, Pulsed-wave Doppler

## Abstract

**Background:**

Although pulmonary artery catheters (PACs) have been the reference standard for calculating cardiac output, echocardiographic estimation of cardiac output (CO) by cardiologists has shown high accuracy compared to PAC measurements. A few studies have assessed the accuracy of echocardiographic estimation of CO in critically ill patients by intensivists with basic training. The aim of this study was to evaluate the accuracy of CO measurements by intensivists with basic training using pulsed-wave Doppler ultrasound vs. PACs in critically ill patients.

**Methods:**

Critically ill patients who required hemodynamic monitoring with a PAC were eligible for the study. Three different intensivists with basic critical care echocardiography training obtained three measurements of CO on each patient. The maximum of three separate left-ventricular outflow tract diameter measurements and the mean of three LVOT velocity time integral measurements were used. The inter-observer reliability and correlation of CO measured by PACs vs. critical care echocardiography were assessed.

**Results:**

A total of 20 patients were included. Data were analyzed comparing the measurements of CO by PAC vs. echocardiography. The inter-observer reliability for measuring CO by echocardiography was good based on a coefficient of intraclass correlation of 0.6 (95% CI 0.48–0.86, *p* < 0.001). Bias and limits of agreement between the two techniques were acceptable (0.64 ± 1.18 L/min, 95% limits of agreement of − 1.73 to 3.01 L/min). In patients with CO < 6.5 L/min, the agreement between CO measured by PAC vs. echocardiography improved (0.13 ± 0.89 L/min; 95% limits of agreement of − 1.64 to 2.22 L/min). The mean percentage of error between the two methods was 17%.

**Conclusions:**

Critical care echocardiography performed at the bedside by intensivists with basic critical care echocardiography training is an accurate and reproducible technique to measure cardiac output in critically ill patients.

**Electronic supplementary material:**

The online version of this article (10.1186/s13089-019-0120-0) contains supplementary material, which is available to authorized users.

## Background

Cardiac output (CO) is the reference standard measurement for assessing target organ perfusion and oxygen delivery in shock. Assessing CO in critically ill patients allows physicians to determine hemodynamic status, identify the most appropriate therapeutic strategy, and monitor the effects of therapy.

Insertion of a pulmonary artery catheter (PAC) has been historically required to calculate CO by thermodilution [1]. However, routine use of PACs in patients with shock is no longer recommended, except in those patients presenting refractory shock, cardiogenic shock, or right-ventricular dysfunction [2]. In recent years, there has been increasing interest to develop non-invasive or minimally invasive technologies to measure CO. Among them, critical care echocardiography (CCE) has emerged as a promising technique that is commonly available, less expensive, and non-invasive (transthoracic echocardiography), or minimally invasive (transesophageal echocardiography) [3, 4].

In stable patients, estimation of CO by CCE has been shown to be accurate when compared to the standard thermodilution technique using a PAC [5–7]. A few studies have compared the accuracy of these techniques in critically ill patients [8], likely due to limited ability to acquire high-quality images in critically ill patients [9]. Despite this, technological advancements are making it easier to obtain high-quality images, and as recommendations on appropriate use of CCE in intensive-care units (ICUs) have emerged [10–13], CCE has become standard practice in many ICUs to evaluate cardiac function.

The primary objective of this study was to compare CO measured by intensivists with basic CCE skills using pulsed-wave Doppler (PWD) vs. PAC in critically ill patients. The secondary objective was to evaluate the inter-observer reliability of PWD-CO measured amongst intensivists with basic CCE skills, as well as identify factors associated with difficult acquisition of PWD-CO measurements with CCE.

## Methods

### Study population

We performed an observational study in a 30-bed medical ICU at Joan XXIII University Hospital in Spain. Approval was obtained from the Joan XXIII University hospital Ethics Committee (IRB # 88/2013), and the study was considered to present minimal risk to subjects. Informed consent was obtained from each subject or their next of kin.

Critically ill patients who required hemodynamic monitoring and were admitted to the ICU were eligible for enrollment from May 2013 to May 2015. Additional eligibility criteria included age > 18, monitoring with a PAC, and interpretable images acquired by CCE. Exclusion criteria included a medical history of congenital heart disease, severe tricuspid regurgitation, severe aortic regurgitation, aortic stenosis, pregnancy, and atrial fibrillation. CO measurements were acquired independent of the subject’s medical and nursing care, and investigators did not change medical management based on findings of this study.

### Training

Before study enrolment, three intensivists were trained to measure CO with a portable ultrasound machine by attending a CCE course that included 10 h of didactics and 4 h of hands-on instruction on acquisition of high-quality parasternal long-axis and apical 5-chamber views. Training also included 10 h of didactics and 6 h of hands-on instruction for advanced cardiac training to learn how to use cardiac software to measure left-ventricular outflow tract diameter (LVOTd) and the left-ventricular outflow tract velocity time integral (VTI).

### Study protocol and data measurements

Subjects were enrolled during the first 24 h of being invasively monitored with a PAC. Decision to insert a PAC was at the discretion of the treating physician. The following demographic, clinical, and physiologic data were collected: age, sex, weight, height, heart rate (HR), central venous pressure (CVP), mean arterial blood pressure (MAP), Acute Physiology and Chronic Health Evaluation II score (APACHE II) [14], the Sequential Organ-Failure Assessment (SOFA) score [15], use of mechanical ventilation (MV), positive end-expiratory pressure (PEEP), use of renal replacement therapy, need for vasoactive drugs, and interpretability of the ultrasound images.

All echocardiographic measurements were done with an Esaote MyLab 30 GOLD cardiovascular ultrasound system (Esaote, Geneva, Italy) equipped with a 3.5 MHz phased-array transducer. Measurements were obtained independently by three blinded intensivists that included a set of hemodynamic parameters with LVOTd, VTI, and HR. All ultrasound images obtained by the three intensivists were stored in digital format and analyzed independently by two blinded investigators to assess the interpretability of the images using a standardized rating scale [16].

Once a subject was enrolled, the three intensivists performed sequential measurements of PWD-CO. The PAC-CO was obtained after each echocardiographic measurement. The PWD-CO was calculated using the maximum value of three LVOTd measurements and the average of three VTI values [17]. The PWD-CO was calculated as follows:

$${\text{PWD-CO }} = {\text{ Stroke volume }}\left( {\text{SV}} \right) \, \times {\text{ HR}},{\text{ where SV }} = \, \left[ {\left( { 3. 1 4 1 6} \right) \, \times \, \left( {{\text{LVOTd}}/ 2} \right)^{ 2} } \right] \, \times {\text{ VTI}} .$$

**Fig. 1:**
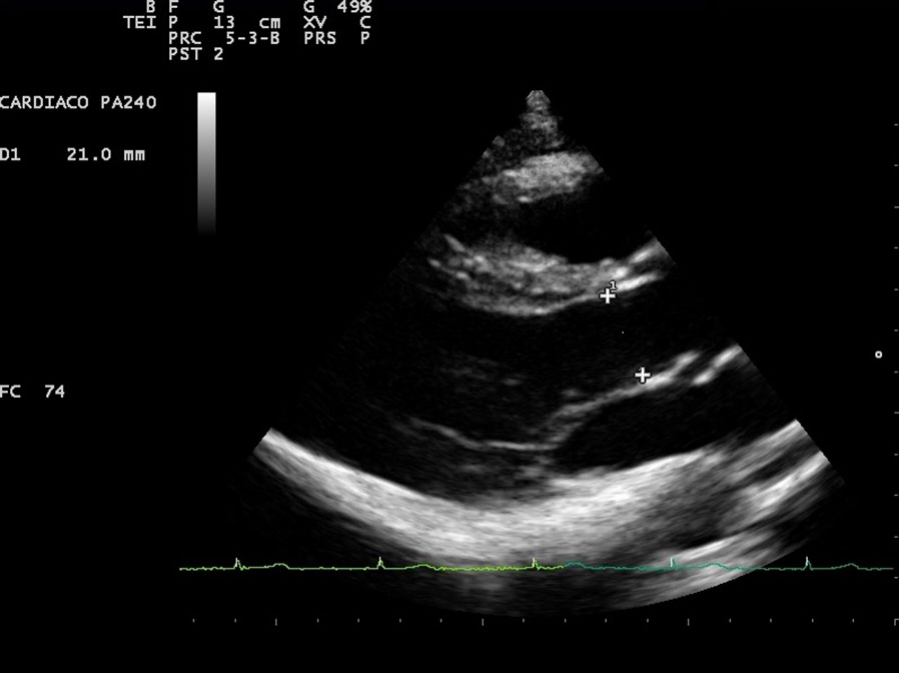
Measurement of the LVOTd from a parasternal long-axis view

**Fig. 2:**
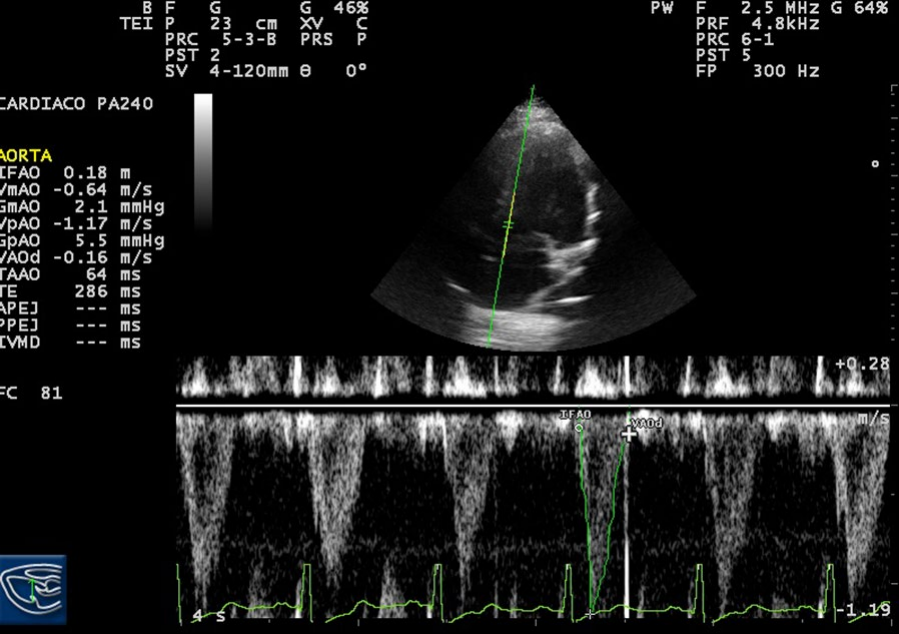
Measurement of the LVOT VTI from an apical 5-chamber view

The LVOTd was measured from a parasternal long-axis view (Fig. 1). The distance from the inner edge to inner edge of the LVOT was measured in a line parallel to the aortic annulus from the base of the right aortic valve coronary cusp to the base of the non-coronary cusp. The VTI was measured by obtaining an apical 5-chamber view and then placing a pulsed-wave Doppler cursor in the LVOT below the aortic valve annulus (Fig. 2). We measured the VTI, at the same time, in the respiratory cycle, ideally at the end of expiration.

The Doppler signal was traced using cardiac software to calculate the VTI, and an average of three measurements was used. The HR was calculated using the ultrasound cardiac software and not by physical examination or telemetry.

The PAC-CO was performed using a 7-French balloon-tipped standard four-lumen PAC model 131HF7 (Edwards Lifesciences Corp, Irvine, CA, USA) connected to a cardiac output monitor LCD medical display-model MOLVL 150-05 (General Electrics, Milwaukee, Wisconsin). PAC-CO measurements were obtained by injecting 10 mL of cold 0.9% saline throughout the respiratory cycle. The CO was measured three times and the results were averaged [18].

All the PWD-CO and PAC-CO measurements were obtained within a maximum of 1 h. The intensivists obtaining the thermodilution results (PAC-CO) were blinded to the PWD-CO measurements and vice versa.

### Statistical analysis

First, a descriptive analysis was performed. Normal distribution of the study variables was confirmed using the Kolmogorov–Smirnov test. Discrete variables were expressed as counts and percentages, and continuous variables were expressed as means with standard deviations (SD) or as medians with interquartile ranges (25th–75th percentile). Differences between groups were assessed using a Chi-squared test or Fisher’s exact test, and Student’s *t* test or Mann–Whitney *U* test, as appropriate. A *p *< 0.05 was considered statistically significant.

The measurement of PAC-CO was considered to be the gold standard measurement for comparison. PWD-CO measurements were compared to the PAC-CO measurements for each individual time-point. Comparisons between these measurements were performed by the linear technique described by Bland and Altman [19]. We defined a clinically acceptable level of agreement between the two techniques when the percentage of error was less than 30% as described by Critchley and Critchley [20]. This cut-off is based on an assumption that a new device destined to monitor CO should have a similar level of precision as the gold standard technique, which in this case is the PAC-CO [21].

The mean differences between the two techniques (bias), the standard deviation (SD) and precision and percentage of error (PE), together with the 95% limits of agreement (LOA) were determined for both techniques. PE for agreement between the two techniques was calculated using the following equation:

$${\text{PE PAC-PWD }} = \, \surd \, \left[ {\left( {\text{precision PAC}} \right)^{ 2} + \, \left( {\text{precision PWD}} \right)^{ 2} } \right].$$

The coefficient of variation (CV) and coefficient of error (CE) were also calculated for both techniques and between them.

The intra- and inter-observer variability was measured by the coefficient of intraclass correlation (CIC) and organized according to the Fleiss kappa scale (Fleiss index). A CIC greater > 0.6 was consider acceptable. Data were analyzed using the SPSS Statistics for windows version 15.0 (IBM corp. Armonk, NY, USA).

## Results

### Patients

A total of 42 critically ill patients were assessed for enrolment in this study. Among them, 14 patients (33.3%) were excluded due to inability to acquire a high-quality image from the parasternal long-axis view to measure LVOTd or apical 5-chamber view to measure VTI. An additional eight patients (19%) were excluded due to atrial fibrillation (*n* = 5), aortic valve disease (*n* = 2), or technical difficulties in obtaining the PAC-CO measurement (*n* = 1).

Data were analyzed from 20 subjects [mean age 67 (± 14) years), 70% males]. Baseline characteristics of the study population are shown in Table 1. Briefly, the most common diagnosis for ICU admission was septic shock (45%). The majority of patients were receiving mechanical ventilation (90%) and vasopressor medications (80%).

**Table 1:** Study population demographics and clinical characteristics

Characteristics	Number of patients (%)
Demographics	
Age, years^a^	67 (± 14)
Sex-male, *n* (%)	14 (70)
Time ICU admission—CO study, days^a^	6 (± 6)
Primary diagnosis, *n* (%)	
Septic shock	9 (45)
Respiratory failure	2 (10)
Surgical	2 (10)
Trauma	1 (5)
Other	6 (30)
Secondary diagnoses, *n* (%)	
DM	8 (40)
Hepatic cirrhosis	2 (10)
COPD	2 (10)
Solid cancer	6 (30)
CRF	2 (10)
Vital signs	
Heart rate, bpm^a^	88 (± 12)
MAP, mmHg^a^	75 (± 9)
BMI, kg/m^2a^	27 (± 3)
Vasopressors and inotropes	
Noradrenaline, *n* (%)	16 (80)
Noradrenaline, mcg/kg/min^a^	0.34 (± 0.24)
Dobutamine, *n* (%)	2 (10)
Dobutamine, mcg/kg/min^a^	5.56 (± 1.71)
Hemodynamics	
CVP, mmHg^a^	13 (± 4)
PAP, mmHg^a^	31 (± 6)
PCWP, mmHg^a^	15 (± 5)
Ventilation	
Mechanical ventilation, *n* (%)	18 (90)
FiO_2_, %^a^	40 (± 10)
PEEP, cmH_2_O^a^	7.78 (± 3.12)
Tidal volume, mL^a^	543 (± 70)
PaO_2_, mmHg^a^	83 (± 15)
PaO_2_/FiO_2_^a^	216 (± 90)
Severity of illness	
SOFA^a^	8 (± 3)
APACHE II^a^	22 (± 9)
Mortality, *n* (%)	5 (25)

BMI: body mass index; DM: diabetes mellitus; COPD: chronic obstructive pulmonary disease; CRF: chronic renal failure; SOFA: sequential organ-failure assessment; APACHE II: acute physiology and chronic health evaluation; MAP: mean arterial pressure; CVP: central venous pressure; PAP: systolic pulmonary arterial pressure; PCWP: pulmonary capillary wedge pressure; PEEP: positive end-expiratory pressure; VAC: vacuum-assisted closure; FiO_2_: fraction of inspired oxygen; PaO_2_: partial arterial pressure of oxygen

^a^Mean ± standard deviation

Compared to included patients, the excluded patients had a faster heart rate and required higher norepinephrine doses. Variables associated with inability to acquire high-quality echocardiographic views were an abdominal wall dressing (*p* = 0.043) and high tidal volumes (*p* = 0.008) (Table 2).

**Table 2:** Factors associated with inability to acquire echocardiographic views

	Possible to acquire			Not possible to acquire			*p*
	*n* (%)	Mean	SD	*n* (%)	Mean	SD	
Age	34	66	13	8	68	19	0.721
BMI	33	28	5	7	29	9	0.631
Mechanical ventilation	27 (77)			8 (23)			0.563
Tidal volume (mL)	26	542	63	7	616	53	0.008
PEEP	26	7	3	8	6	1	0.116
Thoracic drain	7 (87)			1 (12)			0.503
Abdominal dressing	2 (40)			3 (60)			0.043

BMI: body mass index; mL: milliliters; PEEP: positive end-expiratory pressure; SD: standard deviation

### Data measurements

PWD-CO was acquired successfully in 20 patients. To acquire the desired measurements with PWD-CO and PAC-CO, a mean of 54 (± 23) min elapsed to perform a complete examination, from setting up the ultrasound machine for the PWD-CO measurement to acquiring the PAC-CO measurement. For measurement of the PWD-CO alone, a mean of 12 (± 4) min elapsed. The mean LVOTd was 1.92 cm (± 0.13 cm) and the mean VTI was 20.85 cm (± 3.72 cm). The average PWD-CO was 5.22 L/min (± 1.17 L/min), which was less than the average PAC-CO of 6.26 L/min (± 1.96 L/min).

**Fig. 3:**
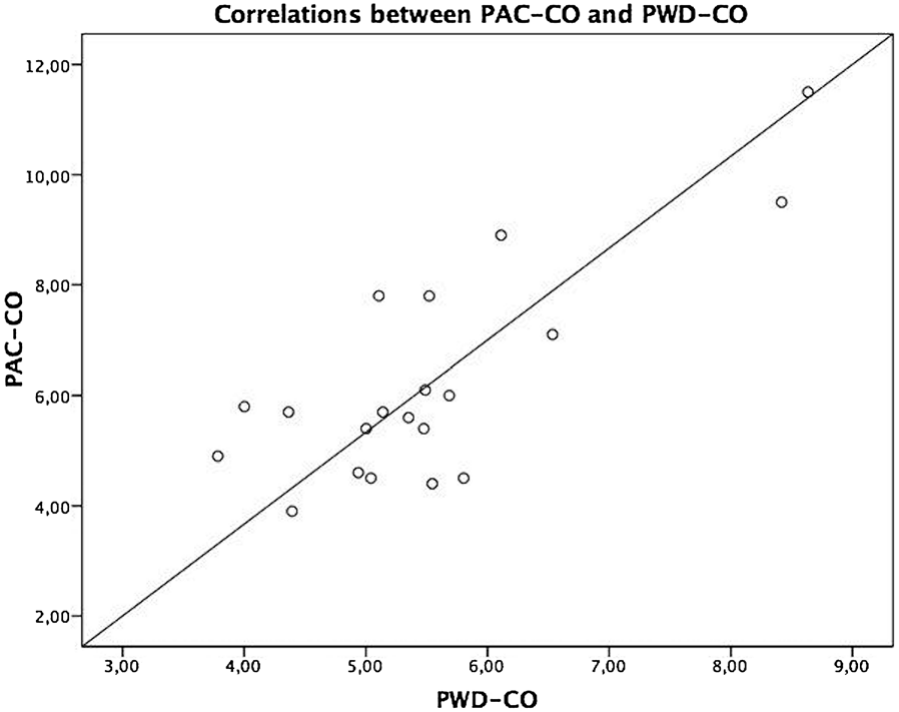
Correlation of PAC-CO and PWD-CO (*r* = 0.78, *p* < 0.0001)

**Fig. 4:**
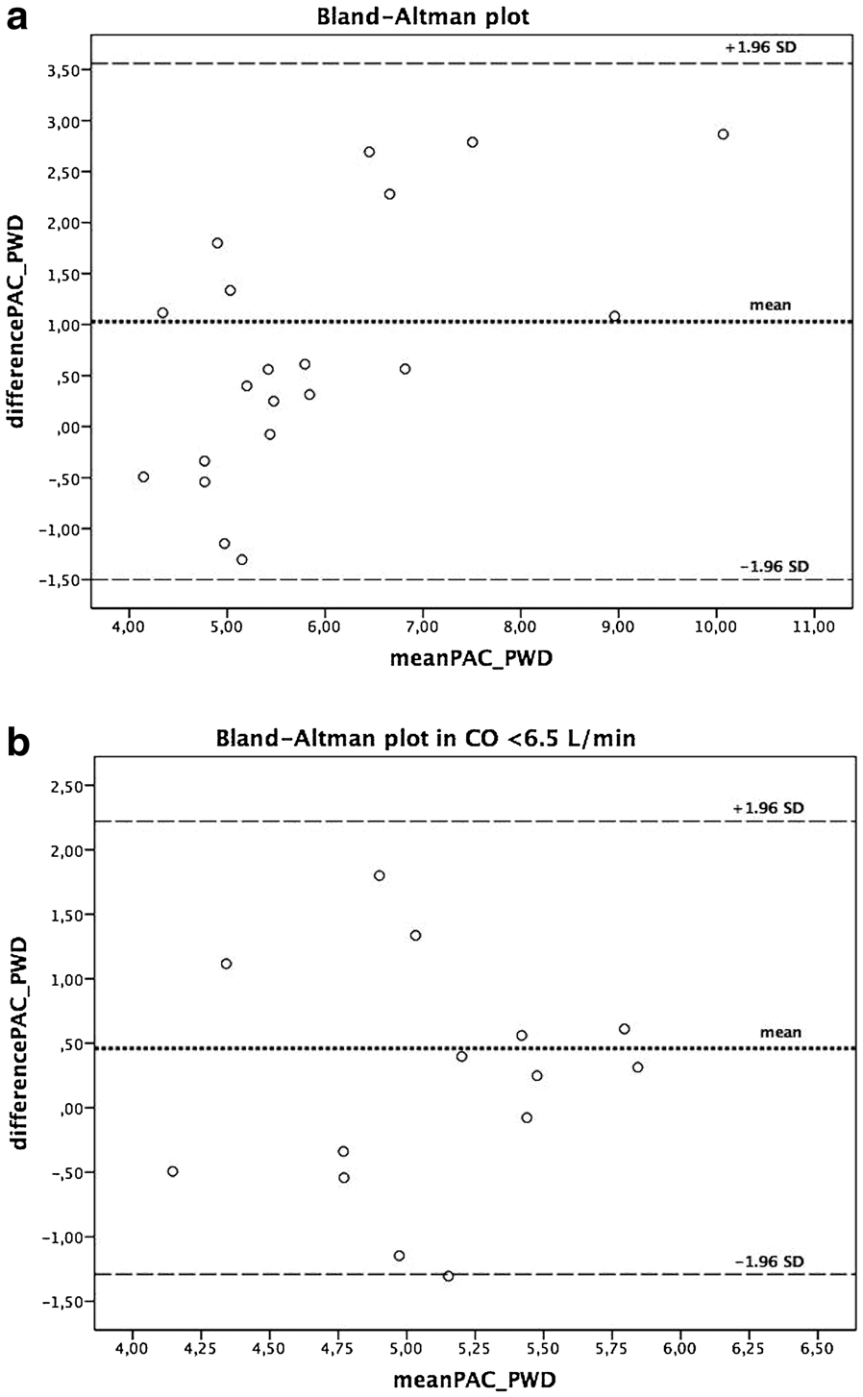
Bland–Altman plots. **a** Difference in PAC-CO and PWD-CO in all patients, and **b** difference in PAC-CO and PWD-CO in patients with CO < 6.5 L/min

Pearson correlation index demonstrated a reasonable correlation between PWD-CO and PAC-CO measurements (*r* = 0.78, *p* < 0.0001) (Fig. 3). To compare CO by both techniques, a Bland–Altman analysis was performed and showed a bias of 1.03 L/min (± 1.27 L/min) with 95% limits of agreement ranging from − 1.50 to 3.56 L/min (Fig. 4). Less difference was seen between both techniques in patients with reduced cardiac output. In those patients with CO < 6.5 L/min, a bias of 0.46 L/min (± 0.88 L/min) with 95% limits of agreement of − 1.29 to 2.22 L/min was found.

The bias, precision, level of agreement, percentage of error, coefficient of variation, and coefficient of error are listed in Table 3. The mean PE between PWD-CO and PAC-CO was 17%. In one patient, the mean PE was higher than 30%. In this case, cardiac rate was normal with a high stroke volume and we could not explain the reason for this outlier.

**Table 3:** Agreement between PWD-CO and PAC-CO

	CO (mean, SD)	Prec.	Bias	95% LOA	PE	CE	CV
PAC	6.26 (1.96)	6%	–	–	–	0.03	0.05
PAC (CO < 6.5 L/min)	5.18 (0.70)	6%	–	–	–	0.03	0.05
PWD	5.22 (1.17)	15%	–	–	–	0.08	0.13
PWD (CO < 6.5 L/min)	4.72 (0.59)	16%	–	–	–	0.08	0.14
PWD-CO vs. PAC-CO	–	–	1.03	− 1.50 to 3.56	17%	0.09	–
PWD-CO vs. PAC-CO^a^	–	–	0.46	− 1.29 to 2.22	12%	0.06	–

CO: cardiac output; Prec.: precision; LOA: limits of agreement; IC: interval confidence; PE: percentage of error; CV: coefficient of variation; CE: coefficient of error; PWD-CO: cardiac output measured by pulse wave Doppler; PAC-CO: cardiac output measured by pulmonary artery catheter

^a^Cohort of patients with CO < 6.5 L/min

Finally, we found an excellent intra-observer and a good inter-observer agreement between the LVOTd and VTI measurements using the Fleiss kappa scale. Detailed results are shown in Table 4.

**Table 4:** Intra- and inter-observer variability

	CIC	95% CI	*p* value	Fleiss index
Intra-observer				
LVOTd				
Observer 1	0.7	0.47–0.85	< 0.001	Good
Observer 2	0.8	0.65–0.91	< 0.001	Excellent
Observer 3	0.9	0.71–0.94	< 0.001	Excellent
VTI				
Observer 1	0.9	0.85–0.97	< 0.001	Excellent
Observer 2	0.9	0.79–0.95	< 0.001	Excellent
Observer 3	0.9	0.73–0.94	< 0.001	Excellent
Inter-observer				
CO	0.6	0.31–0.82	< 0.001	Good

CIC: coefficient of intraclass correlation; CI: confidence interval; LVOTd: left-ventricular outflow tract diameter; VTI: velocity time integral; CO: cardiac output

## Discussion

In this study, we found an acceptable agreement of CO measured by CCE vs. PAC with thermodilution, and the inter- and intra-observer reliability was high. These findings suggest that CO can be accurately measured in critically ill patients by intensivists with the basic CCE training. However, it is important to recognize that high-quality transthoracic images to calculate CO could only be obtained in about half of eligible patients.

Although studies since the 1980s have shown that PWD measurements can accurately determine CO [4–9], a few studies have compared PWD-CO vs. PAC-CO in non-selected, critically ill patients. A recently systematic review of cardiac output measurements by echocardiography vs. thermodilution [22] concluded that the two techniques are not interchangeable. Twenty-four studies of critically ill and non-critically ill patients were included and both transesophageal and transthoracic echocardiography were used in these studies. None of the studies assessed inter- and intra-observer variability. Important limitations of the studies in this systematic review were small sample sizes, heterogeneity, and inadequate statistical analyses.

To our knowledge, one study that compared the use of PWD-CO vs. PAC-CO in critically ill patients found high accuracy and precision between the two techniques [23]. Although the design of this study is comparable to our study, the PWC-CO measurements were obtained by intensivists with extensive experience in CCE.

Similar to previously published studies, our bias analysis showed a systematic underestimation of CO by PDW compared to thermodilution by PAC [24]. This discrepancy was more notable in patients with high cardiac outputs (Fig. 4), probably related to the influence of high flow velocities and turbulent flow over the PWD signal, variability of the VTI angle [25], physiologic fluctuations in stroke volume, and size of the aortic valve orifice [26].

## Strengths and limitations

Our study demonstrated that intensivists with basic CCE training can assess cardiac output in an unselected population of critically ill patients with an acceptable level of agreement between the PWD-CO and PAC-CO measurements. Although isolated CO values should be interpreted with caution, our findings indicate that PWD-CO measurements were accurate over a wide range of cardiac outputs, showing an even stronger correlation in patients with a cardiac output < 6.5 L/min, which can have important implications for the management of vasopressors and fluid therapy.

Additionally, our study is one of the few studies that assessed the inter- and intra-observer variability, and reported the challenges of acquiring high-quality transthoracic images by intensivists with basic CCE training. The intra-observer agreement was excellent and inter-observer agreement was good for ultrasound measurements of LVOT diameter, VTI, and CO. The coefficients of intraclass correlation were acceptable and similar to values described in the literature [27], suggesting that serial measurements, even if performed by different observers with basic training, can be sufficiently reproducible in clinical practice. We also found a significant association between abdominal wall dressings and poor-quality images.

Our study has several limitations. First, the total number of subjects from whom data was analyzed was small (*n* = 20). Approximately half of the patients were excluded due to difficulty in acquiring high-quality images. This limitation of our study is similar to the other studies [28, 29] where high-quality images were not acquired due to use of mechanical ventilation and high levels of PEEP. Furthermore, use of PACs for hemodynamic monitoring has been progressively decreasing in our intensive care unit given the availability of non-invasive methods to measure CO. Thus, use of a PAC was left to the discretion of the attending physician when another less invasive methods of monitoring CO could not be utilized.

Another limitation of our study is the time required to acquire the CO measurements, which averaged close to an hour for a complete examination [mean 54 (± 23) min]. Although this amount of time would be impractical in clinical practice, it is important to note that several measurements were obtained to follow our research study protocol. Most important, the mean time to acquire only the PWD-CO was 12 min, which is realistic to perform in clinical practice. The time and accuracy of these measurements could potentially be improved if acquired by experienced intensivists or cardiac sonographers.

Finally, limited experience of the intensivists in our study was likely an important factor that reduced the accuracy of the PWD-CO measurements. This limited experience is probably due in part to the fact that standards for CCE education currently vary by country, and there is no widely accepted consensus on the training of intensivists [30], despite the recommendations of professional societies to define competencies for basic and advanced training levels [10, 11].

Although an acceptable level of agreement was achieved between CO measured by CCE vs. PAC, the effect of individual or serial measurements of CO on clinical outcomes in critically ill patients is unknown. A recent study found a moderate level of agreement in the hemodynamic assessments performed using transpulmonary thermodilution (TPT) vs. CCE in ventilated patients with septic shock. However, there was no impact in mortality or lactate clearance [31]. Future studies should explore the impact of assessing CO by CCE on mortality and other important clinical outcomes.

## Conclusion

In conclusion, our findings demonstrate that intensivists with basic critical care echocardiography training can accurately and reliably measure CO in critically ill patients compared to gold standard measurements using a pulmonary artery catheter. However, an important limitation is the inability to obtain high-quality transthoracic images to calculate CO in approximately half of eligible patients.

## Additional file

**Additional file 1.** PWD-CO vs. PAC-CO Data.

## Abbreviations

CO: cardiac output
PAC: pulmonary artery catheter
TTE: transthoracic echocardiography
TEE: transesophageal echocardiography
CCE: critical care echocardiography
ICUs: intensive-care units
PWD: pulsed-wave Doppler ultrasound
LVOTd: left-ventricular outflow tract diameter
VTI: left-ventricular outflow tract velocity time integral
HR: heart rate
CVP: central venous pressure
MAP: mean arterial blood pressure
APACHE II: Acute Physiology and Chronic Health Evaluation II score
SOFA: Sequential Organ-Failure Assessment score
MV: mechanical ventilation
PEEP: positive end-expiratory pressure
SV: stroke volume
SD: standard deviations
LOA: limits of agreement
PE: precision
CV: coefficient of variation
CE: coefficient of error
CIC: coefficient of intraclass correlation
